# Extracts and Gleanings

**Published:** 1876-03

**Authors:** 


### Extracts and Gleanings.

---

*Old Frinds w.th New Faces.*—Our forefathers were wont to burn tar and naphtha, and make great fires of wood and coal, and burn sulphur, as a means of disinfecting and purifying the air in times of great plague and sickness; and our foremothers (if we may use such a term without disrespect) set great store by lavender and rose-leaves, and rosemary, and divers aromatic herbs, chiefly of the labiate order, which they kept in their rooms, their linen-presses, wardrobes, cupboards, drawers, and boxes. We younger folks have, perhaps, too quickly condemned these practices as foolish and unmeaning. Chemists have long taught us to recognize sulphurous acid, produced whenever sulphur is burnt, as a powerful disinfectant. Carbolic acid, found in tar, and produced by the combustion of wood and coal, is also one of our most popular antiseptics. But there seems little doubt that we may carry our vindication of our ancestors and ancestresses a little further, and claim for their sweet herbs and fragrant things a higher use than the doubtful one of ministering to the pleasure of the olfactory nerves. Dr. Day, of Geelong, has shown that many substances of the hydrocarbon group, ether, kerosene, naptha, turpentine, and many tinctures, slowly develop peroxide of hydrogen by exposure to light and air. Now, all the labiate family, and nearly all the ordorous substances the men and women of old times were partial to—the ingredients of their pots-pourris and scent vases—contain hydrocarbons which acquire this very property of absorbing oxygen and developing peroxide of hydrogen, which Dr. Day thinks to be identical with the antozone of Schonbein. In his first communication to the *Lancet* (in 1864 and 1865,) Dr. Day imagined that ozone was thus generated, but further experiments, and particularly the reaction with guaiacum resin, have convinced him that these hydrocarbons generate or acquire antozone, or, in other words, the peroxide of hydrogen. In a paper read before the Medical Society of Victoria, on June 4 last, and published in the *Australian Medical Journal* for June, 1873, Dr. Day carries his investigations a step further, and recommends strongly the use of papers, muslins, and other textile fabrics, soaked in kerosene, gasolene, benzine, and paraffin, etc., which have been thus exposed to the air. But the most interesting part of this communication is, first, the indirect evidence of the remarkable immunity from disease enjoyed by workers in petroleum, furnished by Dr. Berry White, Assistant

Surgeon in charge of troops, and Civil Surgeon Dibrooghur, in reference to Makoom, in Upper Assam, where there are petroleum works, for the details of which we must refer to our Australian contemporary; and, secondly, the fact referred to by Dr. Day, of the remarkable permanency of the hydroxyl or peroxide of hydrogen formed in this spontaneous way—some of the sheets of paper having been prepared seven or eight months before, and still giving the reactions of this substance. Dr. Day gives the preference to gasoline, which is almost identical with benzine. He also uses ozonic ether (such as Robbins, of Oxford street, prepares, which is really a solution of peroxide of hydrogen) in the proportion of one part to eight of lard, in cases of scarlatina, as a means of preventing the spread of that disease. The use of lard or sweet-oil for that purpose has long been popular in England, in measles as well as scarlatina, but if it can be shown that the ozonic ether and lard can be kept combined sufficiently long, we think Dr. Day's ingenious plan a decided improvement. Dr. Day informs us, by letter, that fresh animal fats, lard and suet, often contain in their natural state peroxide of hydrogen, loosely combined, which can be demonstrated by manipulation and appropriate tests. One of the Victoria speakers, Dr. Johnasson, thought there might be danger in the use of petroleum papers, on account of their giving off inflammable vapors, but even supposing this to be so, the risk seems to us to be very easily guarded against.—*London Medical Times.*

*The Use of Baths in the Summer-Complaint of Children.*—By J. G. Thomas, M.D.—This disease has been almost epidemic with us this summer, and accompanied with more persistent and obstinate fever than I have ever before known it.

The treatment alone by internal means has been very unsatisfactory; in fact, with me it has always been so in a certain percentage of the cases, for the reason that it is oftentimes impossible to get medicines to remain on the stomach.

In the cases coming under my observation the temperature ranged from 101° to 105°; and it was those of a very high degree of fever that caused me to get in the habit of using what I call the *reduction* treatment by cold water; and I now employ it in all cases of any fever, varying the temperature by the temperature of the patient. Usually by placing a little patient with fever of 102° to 103° or 104°, in water of temperature 70° to 85° for twenty or thirty minutes, the heat will run down to the normal point or below it, and the child will go quietly to sleep, and wake up in an hour or two, much improved. In families where I have used this for the first time, I have found it prudent to stay for an hour or two and see the treatment properly carried out, for many nurses and mothers will at first be afraid of it, but they will soon get over all this fear when they observe the charming effect it has upon the child. * * *

I first take the temperature of the child before it goes in the bath, and then the temperature of the bath, and, as a rule, have the child put into the water at the supposed temperature at which it is in the habit of bathing. But some children are very nervous about going into the water; with such as these we should be very deliberate, and should allow them to get accustomed to it. They should be put into water a little above tepid, to which colder water should be gradually added until the temperature is reduced to 75°; when the fever is very hot, and does not yield readily, to 70°. In many cases the children will scream a short time, but if they are properly managed their cries soon cease, and they wake up an hour after they are taken out, much improved; whereas before, they were unable to sleep for the nervous twitchings and threatenings towards convulsions. The child, after it has been in the bath for from twenty minutes to half an hour, should be taken out, the axillary region dried with a soft towel, and its temperature taken. If we find the heat has gone down to the normal degree or a little below, the child should be wrapped in a light woolen blanket and put in its bed, when, as said above, it will almost invariably sleep for some time. But in one hour its temperature should be taken again, and if it has gone up, the same process should be gone over. Thus I have kept it up in some persistent and severe cases for days, making the child comfortable, and giving time for the effect of other controlling remedies.

In some cases the fever once reduced in this way will not return for four, five, six, or twelve hours, whilst in others it does not return at all; and in all the baths control the heat so as to make the case one of much less gravity, provided they be properly and persistently used.

I have persuaded myself, since the introduction of the clinical thermometer into use, that heat in the blood sometimes causes convulsions, and that there are many cases in scarlatina, for instance, as well as other diseases, which have died from the effece of heat upon the globules of the blood, which we are in ths habit of attribiting to the effect of poison of the disease. Thit conclusion of course is founded alone upon clinical observation, without any pathological investigation. * * *

I have also tried the same reduction treatment in a few cases of malarial fever this season in adults; in these it has acted in the same happy way.

There are scattering cases reported in the journals where it has been tried in rheumatism, scarlatina, etc., with high degrees of temperature, in all of which I propose to try it in future, particularly in scarlatina where the eruption does not appear upon the surface as it should, and the temperature of the patient ranges high.—*Philadelphia Medical Times.*

*The Puerperal Diseases of the Mammæ.*—At the lying-in institution at Dresden, Dr. Huebner has collected some statistics of the most common affections of the breasts in puerperal women. He finds that of 2,300 who were able to nurse, 918 or 39.9 per cent. suffered from effections of the breast. It is aupposed that the results of similar observation in private practice would be more favorable than those made in an hospital. Forty per cent. of those attacked were primiparæ, and the thirty-third year seemed the most liable to such difficulties. The condition favoring those diseases were a strong constitution, a delicate skin, a medium or small size of the breast, a short and retracted nipple—one difficult for the child to get hold of—a bad development of the parenchyma of the gland, and a small quantity of secretion in the gland previous to confinement, as against the opposite state of these facts. The pressure and suction necessarily exercised by the child in the act of nursing determine, as primary affections in the nipple, redness, erythema, vesicles, and fissures, followed secondarily by swelling of the nipple, of the milk ducts, with scabs, erosion and ulceration. Eczma is to be considered as a special disease. In addition to these affections, the areola is liable to follicular abscess and partial inflammations resulting in localized indurations. Inflammation of the parenchyma of the gland, or mastitis parenchymatosa proper, appears to begin as a hyperæmia of the interacinous tissue, with exudation into the meshes of the connective tissue, leading to strangulation and inflammation of the separate acini, the pain being due to pressure on the nerves. These changes are accompanied by increase of temperature and the frequency of the pulse. Wounds of the nipple, blows on the breast, a chill or a powerful mental emotion may, on the second, third or fourth day, convert the physiological swelling of the gland into a mastitis.

Among two thousand and three hundred puerperal women, the author found one hundred and thirty-six cases of mastitis. He states that a previous mastitis does not prevent nursing subsequently, nor does nnrsing often evoke a new mastitis, although marked alterations in the gland and many cicatrices and contractions predispose to it. His treatment of all lesions of the nipple and areola consists in the constant applicatiion, day and night, of lukewarm compresses, wet with lead-water; fissures, ulcers, and excoriations being touched once or twice a day with balsam of Peru, and the breast well supported. The child should nurse less often than usual, and where possible through a nipple shield. He recommends the warm lead water in mastitis also, to be followed by strapping or the breaast and free incision, while suppuration is promoted by poulticing.—*Deutsche Ztschp. f. Med.*, 21 and 22, 1875; *Schmidt's Jahrb.*, Sep., 8, 1875.

*Carbuncle and Felon.*—The following views on the treatment of these affections are given by Dr. C. P. Gage, in the *Transactions* of the New Hampshire Medical Society, 1875:—

"In the treatment of carbuncle, erycipelas and furunculi my treatment has always been based on the theory that they are all blood diseases. After clearing the prima via, the free use of ferri muriate, quinine, and other tonics, together with some stimulants, and good nutritious food, will seldom fail to effect a cure. Local application may afford some comfort, but will do little, if anything, further than that towards a cure.

"In carbuncle, constitutional treatment, with soothing poultices, will do all that can be done to restore in any case. Crucial incisions should never be resorted to. The whole thing is nothing more nor less than an inflammation of the cellular tissue running on to death of the part. When the separation takes place, recovery takes place, if the system does not succumb.

"In the felon the same general plan must be followed, with a free incision of the part, merely with the expectation of procuring ease from the pain caused by the distention."

In the *Transactions* of the Medical Society of the District of Columbia, Dr. Triplett speaks of the efficacy of his treatment of carbuncles, viz: amputation. Hoped every one would try it for himself. What was the objection to it? Was it that we destroyed too much tissue? Carbuncle destroyed its own tissue and took weeks and months to do it. A few days ago he amputated a carbuncle, and the result was so decided that it clearly proved it to be the best treatment. The patient, a negro, aged 40, had a large carbuncle upon the back; the entlre back was œdematous. He had suffered for seven or ten days; 10 grains of quinia and three of opium did not relieve the pain. Dr. Triplett gave chloroform, made a long incision (we could not make skin flaps in the usual way but had to cut the tissues like cheese), and cut out the solid mass afterwards. Hemorrhage was profuse, for these things are wonderfully vascular. Result was, that to-day the patient had no pain, fever gone, appetite good, the man was himself again. Carbuncles take their own time; we should cut them out, and the sooner the better. The hemorrhage was immediate, and in fact, removed from the general circulation; we had no shock. Would not hesitate to amputate, even after sinues had been established. He cited the case of a widow Kelley, of Woodstock, Va., whom he found in a typhoid condition from carbuncle. Her husband had died of the same disease. Feeling that unless relieved she would certainly die, he removed the whole diseased tissue, and the patient made a speedy recovery.—*Med. and Sur. Reporter.*

*Peruvian Bark in Sore Throat.*—Dr. Holden recommends the following formula as exceedingly efficacious in diptheritic sca-r latina and other forms of sore throat:—

R. Corticis peruvianæ flav., ..... .. ...........dr. ij.
Acaciæ pulv. ..........................................dr. j.
Sacch. alb., ............................................dr. ss.
M.

S. Mix one-half of this powder in a tablespoonful of cream, and apply frequently with a camel's-hair brush.

*Pruritus.*—For the intolerable pruritus, common in fall and winter, many physicians use Dr. L. Duncan Bulkley's prescriptions, given in the *Transactions* of the American Medical Association. We repeat them here:—

*Unguentum Anti-pruriticum.*

R. Pulv. gum camphor.
Chloral hydrat. ................................aa dr. j.

Grind well together in a mortar, till they form a fluid, and add slowly, simple cerate, one ounce.

*Liquor Picis Alkalinus.*

R. Potass. causticæ ................................dr. j.
Picis liquidæ........................................dr. ij.
Aquæ..... ............................................oz. v.
M.

Dissolve the caustic potass. in the water, and add gradually the tar, mixing them well in a mortar. Use in solution with from 8 to 16 parts of water.

*Treatment of Certain Tumors by the Subcutaneous Injection of Alcohol.*—Schwalbe, of Weinheim, has reported one hundred cases of various forms of indolent glandular swellings treated successfully by the subcutaneous injection of the tincture of iodide. Latterly he has used injections of simple alcohol in fifty similar cases, and has found the results equally favorable, and the time required for a cure no greater, and he therefore concludes that the alcohol is the essential remedial agent. He explains its curative action as follows: It establishes a state of chronic inflammation in the connective tissue, causing it to contract by degrees, and thus pressure is brought upon the vessels and the lymphatics are obliterated. These effects, and the consequent hardening of the connective tissue, he proposes to utilize in the treatment of other tumors, and he reports the cure of fatty tumors by the use of such injections, to which some ether was added in order to dissolve the fat. He finds, however, the most important application of his plan in the treatment of cancer, by preventing its extension to the neighboring tissues and lymphatic glands. The tumor is first to be isolated, as it were, by causing the connective tissue on all sides of it to become shrivelled; then, the contracting connective tissue, approaching the growth itself, dresses upon it, cuts off its blood supply, and so causes it to disappear by atrophy. Lymphatic glands which are already affected are to be similarly treated. Schwalbe, with Dr. Hasse, claims to have cured three cases of cancer of the breast in this way.—*Sitzungsbericht der niederrhein, Gesellsch[illegible]ft in Bonn*, 1874. —*Allg. med. Central Ztg.*, Jan. 23, 1875.

*Treatment of Anal Vegetations.*—M. Cruveilhier, of the Hopital St. Louis, believes, with the majority of surgeons, that these sessile or pediculated vegetations are not dependent on the symphilitic virus, and that therefore internal treatment is not appropriate. They must be destroyed locally in a radical manner, for if a single one is allowed to remain, however small it may be, it will form the starting-point of new vegetations, which will multiply without measure. He has employed excision with curved scissors, followed or not by cauterization with diluted perchloride of iron. But he prefers a milder and often more efficacious remedy, pure chromic acid, which, applied to the small tumors, the healthy parts being protected, mumifies them and causes their decay. This caustic is less painful than nitric acid, or acid nitrate of mercury, and less often causes inflammation in the neighborhood. If these means fail, the operation of removal with the knife must be resorted to.—*Revue de Therap.*, 15, 1874.

*Ipecac in Dysentery.*—Dr. E. H. Fournier stated that in the last few weeks he had been enabled to enlarge his experience in the treatment of dysentery by ipecac. One case, a colored woman, who had not been under the care of any physician, had been suffering about a month. There was great tenesmus straining and constant desire to go to stool, together with the characteristic discharge, frequently repeated. Gave twenty grains of ipecac, preceding it by a full opiate in the morning, and repeated at night. Next day she was entirely relieved of the griping, straining, etc., evacuations larger and fœcal, and she expressed herself as feeling well. There was still, however, considerable tenderness on pressure over the abdomen. She had no further treatment till the third day, when, there being some tendency to relapse, she had some slight treatment. This was followed by tonics, and she soon left the hospital cured.

He also related a second case, in the person of a young Frenchman, also an inmate of the city hospital, who had been suffering from dysentery nearly a month—sometimes better and sometimes worse. On admission, he was suffering a great deal, with all the symptoms of acute dysentery. Treatment same as that detailed above, and followed by equally satisfactory results.

Dr. Fournier remarked that he sometimes gave the ipecac, combined with opium, in the proportion of three or four grains of the former to one of the latter. He always administers the ipecac in pill or bolus, if the patient can swallow it. He prefers this method to giving the powder in substance, as it is less liable to nauseate. He is treating at this time some cases of chronic diarrhea with pills containing small doses of ipecac and opium, and apparently with the effect of gradually benefiting the patient.

Dr. Read had used the ipecac treatment in a number of severe cases recently, and with the happiest results. One case in particular—a young physician at State Line, Mississippi, who in alarm had sent for him to attend him, the disease having appeared in that locality in rather a violent form, with several fatal cases. The patient was cured with three doses of ipecac, ten grains each, preceding each dose with an opiate.

Dr. F. A. Ross (the President) remarked that it was a curious fact of medical history, that though ipecac was first introduced to the profession as a remedy for dysentery, and was very extensively used in Europe and elsewhere for this purpose, yet from some cause or other it gradually fell into disuse in this connection; and now, following a very long period of almost complete abandonment, we are again hearing of its employment frequently in this disease. He said that pills were undoubtedly the best way to administer the remedy, as less likely to induce nausea and vomiting, which would frustrate the plan of treatment.—*Chicago Medical News.*

*Properties of Grindelia Robusta.*—Dr. Henry M. Fiske has employed the grindelia robusta, an herbaceous perennial plant, a native of the west coast of America, in a variety of cases with excellent results. It is a demulcent as well as stimulant, and makes an excellent dressing for vesicated surfaces. In burns, the fresh herb bruised and applied frequently over the injured parts is an unequalled anæsthetic. It is an excellent remedy in uterine catarrh, or in catarrh of the genito-urinary tract. In subduing the intense burning and itching of vaginitis, as well as painful priapism, it is of great value. In the first, the tincture or fluid extract, of the strength of one tablespoonful of either to about four tablespoonfuls of water, should be used as an injection three or four times a day, and cloths should be soaked in it and applied to the pubes as hot as can be borne. In the other, a direct application should be made of the bruised plant, in the form of a poultice, if possible, changed frequently. In a few hours marked beneficial results will be noticed. In iritis, no matter what the cause, whether the gout, rheumatism, scrofula, or violence, it seems to be almost a specific. Dr. Fiske gives two cases of iritis in which he employed grindelia with excellent effect.—*Pacific Medical and Surgical Journal.*

*The Hypodermic Use of Apomorphia as an Emetic in Children.* Dr. William F. Duncan, after considerable experience with apomorphia, says that the qualities which recommend it particularly are the rapidity of its action, the absence of danger from an over-dose, the lightness of its secondary effects, the shortness of the period of nausea, the easy manner of its introduction.

The average time at which emesis has occurred, after the introduction of this drug under the skin, is 2.9 minutes, which is very much less than the shortest time noticed when using the yellow sulphate of mercury.

The longest time for emesis to appear was 4.15 minutes, in a case of alcoholism, while the shortest was 1.75 minutes, in a case of capillary bronchitis.

The dose of apomorphia, hypodermically used, for an adult, ranges from gr. $\frac{1}{10}$ to gr. $\frac{1}{20}$, but in children it is quite large in proportion.

| | | |
|---|---|---|
| For a child of | 18 months | gr. 1-50. |
| " " | 2 years | gr. 1-40. |
| " " | 3 " | gr. 1-35. |
| " " | 5 " | gr. 1-30. |
| " " | 8 " | gr. 1-25. |

Glycerin seems to preserve the strength of the drug, and alcohol will dissolve it more readily than water; so that it should be prepared after the following formula:

R.—Apomorphiæ........................gr. vii.
Spt. rectificat........................*m* xx.
Glycerin........................*m* x.
Aquæ........................*m* l.—M.

—*Medical Record*, August 7, 1875.

*Chloral Suppositories.*—The production of a chloral suppository containing a sufficient proportion of this drug to cause sleep has heretofore been deemed impossible. M. H. Mayet, pharmacien, of Paris, has, however, devised the following formular, by which he manages to get forty-five grains of chloral in each suppository:

R.—Ol. theobromæ........................gr. xxv.

Cetacei,

Pulv. chloral, aa........................gr. xlv.

For one suppository.

These suppositories are of good consistence, and may be easily put into use.—*The Druggist's Circular.*

*Pernicious Anæmia.*—Dr. Biermer, of Zurich (see "Translations"), gives the name of "progressive pernicious anæmia" to a disease which he believes to differ essentially from other forms of anæmia. The subjects of it become exceedingly pale, and the skin of their hands, feet, and face, acquires a swoolen appearance, but there is no perceptible decrease of the fat covering the body. Ecchymoses appear in the retina, though vision remains intact. There are probably small hæmorrhages in the brain-substance. The disease is said to be always fatal. Dr. Biermer has seen fifteen cases during the last five years, the majority being women. The symptoms resemble somewhat those of albuminuria, but the urine contains no albumen. Necropsies have invariably shown partial fatty degeneration of the papillary muscles of the heart, and fatty degeneration of the small blood-vessels of various organs.—*N. Y. Med. Jour.*

*Cerebro-Spinal Fever.*—Dr. J. B. Hamilton says: I am not much in favor of set formulæ, but should I be called to treat a case to-day, I should outline the treatment as follows: For the first twenty-four or forty-eight hours—

R.—Ext. ergot, fl. one ounce.
Sp. ammon. arom., two ounces.

A teaspoonful in a little water every four hours.

R.—Potass. acetat., twelve drachms.
Aqua camp., fl. six ounces.

A tablespoonful every two hours until diuresis is produced. In addition to these remedies I should direct a warm bath, followed by wrapping in flannel or rubbing with dry mustard, every three, four, or six hours, according to the urgency of the case. Stimulants may be necessary from the outset.

As soon as the syptoms show any amelioration, the ergot-mixture may be diminished in frequency, at the third or fourth day discontinued. Quinine in large doses will then be found of advantage, and a more stimulant diuretic, as spts. etheris nit., may be substituted for the potassa acetate; and for the sequlæ nothing in my hands has proved more serviceable than iodide of potassium as occasion demanded.—*New York Medical Jaurnal.*

*Method of Instantaneously Arresting Palpitations of the Heart.*—Dr. J. Lardies describes (*L'Union Medicale*, Aug. 21) a method by which he asserts that palpitations of the heart, not due to any material lesion of that organ or of the nervous centres, may be instantaneously arrested. He discovered this means accidentally, when troubled himself with palpitations. Since then he has directed many of his patients suffering from this trouble to bend their body, the head down, and the arms hanging so as to momentarily cause congestion of the upper part of the body. In all cases of nervous or anæmic palpitations the heart quickly resumed its normal action. He adds that if, while in the above described position, respiration is arrested for a few seconds only, the relief afforded is still more speedy.—*Clinic.*

Ergot of Rye as an Antipyretic.—M. Hayen reports, in the *Revue de Therapeutique*, a trial of ergot of rye in cases of enteric fever, with the object of lowering the temperature. The results he has obtained have been very satisfactory, and its employment in this disease seems to him preferable to that of sulphate of quinine or of digitalis. Under the influence of ergot there is much more rapid defervescence; and at the period of the acme, instead of there being a rise in the temperature chart, a plateau is obtained. In some cases in which the ergot was only given during the day, the evening temperature was not so high as the morning. The dose varied from thirty to fifty grains in the twenty-four hours.

*Purpura Hæmorrhagica.*—Dr. B. W. Richardson delivered an address on this subject before the Medical Society of London, at a meeting held November 9th. He divides the disease into three principal classes, and points out the difference in the cause, diagnosis and treatment of each. In what he terms the *aqueous* variety, the water of the blood is in excess, and the colloids relatively diminished; in the *saline* class the colloidal element is held in undue solution by excess of saline substances; in the *vascular* form of purpura the disease is due to some degenerative change in the capillaries, facilitating rupture or transudation. These different forms of the disease are believed by Dr.

Richardson to be characterized by different forms of eruption. The paper will be found in full in the *Medical Times and Gazette* of November 28.

*Treatment of Chronic Ulcers of the Leg.*—For the treatment of these ulcers, which are of such frequent occurrence among the poorer classes, Dr. Bidder recommends the starch-bandage. The ulcer and leg, having been cleaned, are covered with a layer of wadding, and then the limb is enveloped in a starch-bandage. After the dressing has dried, the patient can follow his occupation. After from five to eight days the dressing is removed and renewed. If the ulcer has callous edges, it is best to strap it first. After treatment of several months in the above manner, the author has cured large and very chronic ulcers.—*Med. Chir. Centralblatt,* 31. 1874. E. F.

*Summer Complaint.*—Dr. A. Jacobi states as follows:—"It comes from over-feeding and hot and foul air; never from teething. Keep doors and windows open. Wash your children with cold water, at least twice a day, and oftener in the very hot season. When babies vomit and purge, give nothing to eat or drink for four or six hours, but all the fresh air you can. After that time, yon give a few drops of whisky in a teaspoonful of ice-water, every ten minutes, but not more until the doctor comes. Where there is vomiting and purging, give no milk, give no laudanum, no paregoric, no soothing syrup, no teas."—*St. Louis Med. and Surg. Jour.*

*Tincture of Chloride of Iron for Nasal Polypus.*—Dr. G. Troup Maxwell (*Phil. Med. Times*) injects into the nostril one drachm tincture of iron, diluted with one drachm water, holding the head back so that the liquid shall come in contact with the polypus. This he repeats daily, with the effect of producing a complete cure in from four to seven or eight days. It causes but slight pain, and does not prevent the patient from attending to his business.

*Electricity in Jaundice.*—The treatment of jaundice by electricity has often proved successful. The procedure is certainly worthy of trial. The position of the gall-bladder is first found out by percussion, and to this point the electrode of an inductive electric machine is applied. The other electrone is placed on the opposite side of the abdominal wall. When the current is passed, a gurgling sound may be heard, and the cure is often immediate.—*Pacific Med. and Surg. Jour., July,* 1875.

*Tepid Baths in Fevers.*—Dr. Berthomier demonstrates that, contrary to the opinion of Ziemssen and Leibermeister, prolonged tepid baths lower the temperature. After noting the favorable effects of tepid baths in tuberculosis, a series of experiences is given, which demonstrate that with baths at a temperature of 36 to 37 degrees C. the thermogenesis of the body is lowered by one or two degrees, and the pulse is lowered by several beats. According to this writer, the depression is more prolonged than that produced by cold baths.—*Bull. Gen. de Therap.*, and *Gaz. Med. Ital., Ven.*, 35, 1874. G. R. C.

*Mercurial Ointment in Boils and Carbuncles.*—Dr. T. Roth lauds, in the *Deutsche Klinik*, local application of gray ointment in boils and carbuncles, especially the early stages. He anoints the affected part with the ointment four times daily, and thereby reduces the inflammation and "backens" the boil most satisfactorily.—*The Medical and Surgical Reporter.*

*A Cure for Bright's Disease.*—Dr. Hegswald says, a half pint thrice daily, of a fresh infusion of the leaves of *Asplenium Scolopendrium*, L., is a most successful treatment in Bright's disease. This is the harts-tongue or spleenwort, and is said to be popular in Devonshire, and elsewhere, for its medicinal virtues. *The Medical and Surgical Reporter.*

*Vegetable Acids.*—From the pen of A. Jacobi, M.D.:—"Nursing women are often forbidden to eat acids, or acid fruits, lest the milk should turn sour. Vegetable acids, as soon as they are taken up into the blood, are converted into alkaline carbonates, long before they could reach the milk glands, therefore, their acidity is destroyed. Instead of being avoided, fruits are extremely desirable as articles of food for the nursing women.—*St. Louis Med. and Surg. Jour.*

*Treatment of Chronic Constipation.*—The *Medical Times and Gazette* contains an interesting article on the "Therapeutics of Chronic Constipation," by Dr. J. K. Spender, of London, and though not of very recent date, we subjoin an extract as presented in the *Half-Yearly Abstract of the Medical Sciences*, relating to the treatment of this annoying malady. The treatment promises not mere relief, but final cure, and "comprises four therapeutic factors: (*a*) minute and frequent doses of watery extract of aloes, very rarely of extract of colocynth; (*b*) a dose of sulphate of iron (gr. jss or ij), always combined with each dose of the direct aperient; (*c*) regulation of the diet; (*d*) constitutional exercise. The author writes chiefly of factors (*a*) and (*b*). The quantity of extract of aloes, in all but extraordinary cases, he says, should not exceed one grain. It is conveniently given in the form of a pill. With this pill there should always be mixed a dose of sulphate of iron varying from one to three grains; this is the essential point of the treatment. Any other tonic of the neurotic kind cannot supply the place of the iron; iron is not only *facile princeps*, but is not interchangeable by anything else. Extract of nux vomica may be added, if the prescriber pleases, as an ornamental appendage or as a means of blending the other constituents together; and belladonna is a remedy of definite auxiliary power; but both these drugs, *quoad* constipation of the bowels, are uncertain or unsatisfactory, and rarely do permanent good. Dr. Spender begins, then, by desiring an adult patient to take a pill composed as above three times a day, immediately after the principal meals. He is cautioned that at first there will be probably no apparent effect, and that two or even three days may pass before any medical evacuation of the bowels takes place, perhaps even then difficult and discomforting. But within the next forty-eight hours there will be most likely an evacuation of the bowels once or possibly twice in the day; *but nothing approaching to purgation ought ever to be permitted*, and, therefore, the patient must be instructed, on the occurrence of the first loose motion, to withhold a pill, and to take only one in the morning and one in the evening. He then continues for a time his morning and evening pill, and is pleased to discover that so slender a medicament has such a decided effect. Not improbably, at the end of another week or fortnight, he is compelled, by the same reason as before, to drop another pill, and the same result is now brought about by one pill daily, as was originally produced by three pills. Within another month, he may reduce his allowance of medicine to a single pill once or twice a week; and finally his whole scheme of medical treatment becomes merely preventive in its design and scope, and he takes a pill occasionally for the sake of maintaining health and warding off old troubles.

"When there is real or fanciful difficulty in the administration of pills, the best way of carrying out the plan above described is by combining the *mistura ferri composita* with the *decoctum aloes compositum*, the doses being determined by the application of the same principles."

This treatment seem, altogether rational, and we hesitate not to recommend it. The object sought is not mere evacuation of the bowels, but restoration of lost tone. A. A. L.

*Sexual Hyperchondriasis.*—Every physician has had greater or less experience with patients suffering, either really or in imagination, from sexual irregularity, which gives rise, in some cases, to a great deal of anxiety and trouble, generally needless. Under these circumstances the physician is sometimes called upon to decide questions that involve *ethics*, as well as therapeutics. We are glad to have the high authority of Sir J. Roget, as a precedent in such cases, and it affords us pleasure to quote the following from a late lecture, published in the *British Medical Journal.* In refering to the case of sexual hypochondriacs, Roget says:

"To all alike you may try to teach a judicious carelessness about these things; a state of mind which would be an inestimable blessing to many besides these sexual hypochrondiacs. Many of your patients will ask you about sexual intercourse, and some will expect you to prescribe fornication. I would just as soon prescribe theft or lying, or anything else that God has forbidden. If men will practice fornication or uncleanness, it must be of their own choice, and on their sole responsibility. We are not to advise that which is morally wrong, even if we have some reason to think that a patient's health would be better for the wrong-doing. But in the case before us, and I can imagine none in which I should think differently, there is not good enough for so much as raising a question about wrong-doing. Chastity does no harm to mind or body, its discipline is excellent; marriage can be safely waited for; and, among the many nervous and hypochondriacial patients who have talked to me about fornication, I have never heard one say that he was better or happier after it."

We reproduce the above, not so much for the scientific truth it contains, as for the excellent moral lesson it teaches. Emenating from such a source, it carries weight with it.

A. A. L.

*Dilatation of the Servix Uteri in Dysmenorrhœa.*—Dr. John Ball recommends the following method of procedure in cases of constricted cervix uteri. Having procured the thorough evacuation of the bowels of the patient, place her upon her back, with the hips near the edge of the bed, and when she is profoundly anæsthetized introduce a three bladed, self-restraining speculum; seize the os uteri with a double-hooked tenaculum, draw it down toward the vulva, and then introduce a metal bougie as large as the canal will admit, following it in rapid succession by others of larger size, until one is reached which represents the size of the dilator. Then insert the dilator and stretch the servic in every direction until it is enlarged sufficiently to admit a No. 16 bougie, which is all that is generally necessary. Then intoduce a hollow gum-elastic uterine pessary of about that size, and retain it in position, by a stem secured outside the vulva, for about a week, in which time it has done its work and is ready to be removed. During this time the patient should be kept perfectly quiet, and usually upon her back. Dr. Ball claims that the operation saves a great deal of time, causes much less constitutional disturbance than the use of tents, and is not only safer than the metrotome, but is free from some serious objctions to the use of the latter, there being no resulting cicatrix to interfere with the dilatation of the parts, and the condition of the patient after an unsuccessful operation being no worse than before. He says that it relieves the constriction entirely, by breaking up all the adhesions, which are often firm and unyielding; that, acting as a derivative, it cures the hyperæmia of the cervix; and that, further, it establishes a radical change in the nutrition of the whole organ.

He details nine cases of stricture of the os and cervix complicated with viginismus, chronic endo-cervicitis, version, sterillity, dysmenorrhœa, etc., in all of which very great relief or permanent restoration to health was effected by rapid and forcible dilatation. In a foot-note the editor of the *New York Medical Journal* quotes Dr. Ellinger, of Stuttgart, as recommending the operation—1, in stricture of the cervical canal; 2, stenosis due to flexions; 3, metrorrhagia in a flabby, swollen uterus, but without new growths; 4, retained catharral secretions; 5, for exploration of the uterine cavity; 6, replacement of a flexed uterus; 7, sterility. Dr. Ellinger declares that he has never had reason to regret rapid dilatation, and urges it, where dilatation is justifiable at all, to the exclusion of all other methods.

*A New Medicine.*—At the last annual meeting of the Medical Society of Virginia, Dr. W. F. Barr called the attention of the fellows of the society to a *new* preparation of iron and alum, manufactured in Washington county, Virginia, from the waters of "Seven Springs." It is made by evaporation, and the analysis, by Prof. I. W. Mallet, finds it to consist chiefly of iron alumina, magnesia and lime. This medicine had been prescribed by the physicians of the southwestern section of the State, and found to be a most excellent tonic and alterative. One advantage it has over the ordinary ferriginous preparations is that it does not constipate the bowels. On the contrary, it acts as an aperient. It had been indorsed and recommended by the Abingdon Academy of Medicine "as a valuable contribution to Materia Medica." He hoped that the fellows of the society, and physicians generally, would give it a fair trial. It is beneficial in dyspepsia, chronic bronchitis, neuralgia, rheumatism, nervous and sick headache, chronic diarrhœa and constipation, leucorrhœa, amenorhœa, dysmenorrhœa, menorrhagia, anæmia, chlorosis, choræ, diseases following intermittent fever, and in all cases in which it is desired to improve the impoverished condition of the blood.—*Transactions Medical Society of Virginia, Oct*, 1875.—*Virginia Medical Monthly.*

*Cod Liver Oil and Lacto-Phosphate of Lime.*—Edward Chiles, (*American Journal Phar.*), says this remedy is being quite extensively prescibed by physicians, and as considerable inquiry has been made as to an eligible mode of prescribing it, I will give my experience in the manufacture of the article, and also a simple process for making syrup of lacto-phosphate of lime.

For a long time I have had demand for a tasteless cod liver oil, and have been in the habit of preparing it in the form of an emulsion with gum arabic water, and covering the odor with a few drops of essential oil of bitter almonds.

Over a year ago I found physicians were prescribing cod liver oil and lacto-phosphate of lime, and I devised a formula for it, based on my experience with the simple emulsion and the syrup of lacto-phosphate of lime, for which a considerable demand had sprung up. The formula I then devised has been followed by me up to the present time, and has invariably given satisfaction, and produces an article which does not separate or become rancid.

I think, however, it should be prepared extemporaneously as prescribed by physicians, and I have not kept it on hand, but prepare it as wanted, thus always giving a perfectly sweet article.

Take of Gum arabic..............................oz. ij drm. ij.
Water......................................f oz. ij.
Syr. lacto-phosphate of lime.......f oz. vi.
Cod liver oil...............................f oz. viij.
Essential oil bitter almonds......six drops.

Rub the gum, water and syrup together, until a smooth mucilage is made, then add the oil gradually with constant stirring, and, lastly, the oil of bitter almonds.

Thus made, each tablespoonful of cod liver oil and lacto-phosphate of lime contains four (4) grains lacto-phosphate of lime and 50 per cent. of cod liver oil. The gum in the above should be selected, ground and passed through a seive of 60 meshes to the inch. Cod liver oil and lacto phosphate of lime, prepared in this manner, forms a preparation free from unpleasant taste and odor, and enables the practitioner to administer these valuable remedies without repugnance on the part of the patient.

*Improved Formula for Camphor Water.*—Wm. B. Addington, Norfolk, Virginia, (*American Journal of Pharmacy*) suggests the following formula:

R.—Camphoræ .............................. dr. iv.
Magnes. carb.............................. dr. ii.
Aquæ destillat.............................. O iv.
Alcohol.............................. q. s.

Take just enough alcohol to dissolve the camphor and bring it to a liquid state; while liquid, add the magnesia and triturate (during this time the alcohol will evaporate.) Then mix the water, as usual, and filter. By making a perfect solution of the camphor, the particles are thoroughly divided, whereas, by the U. S. P. process, only enough alcohol is added to break up the adhesion of its particles and reduce it to powder, and all must have noticed the numerous small grains of camphor left on the filter by the present process. Camphor water is made by the process I suggest in one-half the time; magnesia is saved by it, and all the camphor directed is taken up in the solution. By the present process it is not. There is no deposit formed on the bottom or sides of the jar by standing. I have tried this formula for the last eight months, and am very much pleased with it.

*An Instrument for the Removal of Retained Placenta.*—Dr. Adolph Rasch, at the last meeting of the British Medical Association (*Obstet. Journal of Great Britain*), advocated mechanical procedures for removing the ovum in those cases of abortion in which ergot and cold fail to arrest the hemorrhage, and the tampon has been given a fair trial without bringing away the contents of the uterus. In most cases this can be done by the fingers in the vagina, aided by outward fixation of the uterus.

But still, cases occur where the retained placenta can be touched but not brought down, and where prolonged and dangerous hemorrhage makes a speedy removal imperative. The instruments contrived for that purpose seem to Dr. Rasch to all have the fault in common that the operator does not feel what he has hold of. His instrument is a sensitive forceps, one-half of which consists of the index finger, the other half of a scoop, with a finely-toothed bowl just large enough for the tip of the index finger, on which it is to be introduced through the os. The scoop is pushed up on the outer side of the placenta, the index finger guiding, and at last pressing the latter into the bowl. Three fingers of the same hand perform, by pressing the stem into the hollow of the hand, what is necessary to transform this single blade, or half forceps, into a complete one. Thus all danger of injuring the uterus is obviated, and a firm purchase of the placenta or membranes effected. After five years' trial, Dr. Rasch warmly recommends his simple and cheap instrument.

*Treatment of Hemiplegia, by Large Doses of Bromide of Ammonia.*—By DR. RIOPEL.—In giving his experience in the use of bromides, the Doctor premises his statements with the remarks, that he is aware that he is liable to strong criticism, nasmuch as it varies from the standard treatment, and especially that advocated by Brown-Sequard and other noted men—viz., ergot, belladonna, strychnia, etc. He reports cases showing the favorable result is treatment, one of which I copy as a typical case.

*The Theapy of Leucocythæmia.*—In his preliminary note to the *Centralblatt fur die medicinischen Wissenschaften*, Dr. Ordenstein states that hereditary syphilis is a prolific cause of the affection. In one obstinate case under his care, in which every kind of treatment had proved futile, he discovered a syphilitic history in the patient's father. A corresponding treatment was now instituted, and the result was a speedy cure. He promises to discuss this subject more fully in another article.—*The Clinic.*

Citric Acid in the Treatment of Cancer.—In *The British Medical Journal* of November 27th, John H. Wood, M. D., reports a case of cancer of the œsophagus and cardiac orifice of the stomach, in which the symptoms were, for a time, very much relieved by the use of citric acid in large doses, combining it, on Dr. Sidney Ringer's plan, with wine of ipecac in minim doses.

Removal of Foreign Bodies from the Ear.—Dr. John Cleland suggests that, in removing foreign bodies from the ear, the point of the probe or needle used for extraction should be placed below the object to be dislodged. By so doing it is placed between two inclined planes, and is readily and easily expelled.—*Phil. Med. Times,* Jan 23, 1875, fr. *The Lancet,* Dec. 5, 1874.

Freckle Lotion.—Take:

| | |
|---|---|
| Citric acid | 3 drachms. |
| Rose water | 12 fl. ounces. |

To apply both of those lotions it is only necessary to moisten a sponge or the fingers with them, and to wet the skin by gentle rubbing.—*Canada Medical Record.*

Treatment of Itch.—It is well-known that frictions with sulphur ointment may render much worse the various inflammatory affections of the skin which are excited by the presence of the acarus. To avoid this complication, the patient should use one part of styrax to two of oil of sweet almonds or olive oil. By this means the acarus is quickly destroyed, and the skin hardly irritated.—*Lancet.*

Pruritus Treated with Vinegar.—Dr. Thackeray, of Dacota, writes to the *Medical News* that he treats the pruritus formicans of pregnancy with cider vinegar, topically applied, and that he has never failed to cure with it. He procured the prescription from the late Prof. Henry S. Dickson.

Chloral in Nocturnal Emesis.—In *L'Imparciale*, March 17th, Dr. Ademolo relates four cases which were completely cured by chloral hydrate. It may be given at first in doses of seven or eight grains at night, gradually increasing the dose to twelve grains.—*American Practitioner.*

